# Primary aldosteronism beyond laterality: integrating subtype assignment with outcome-oriented risk stratification

**DOI:** 10.3389/fendo.2026.1842927

**Published:** 2026-05-19

**Authors:** Shida Chen, Haifeng Xu, Chengan Xu

**Affiliations:** 1 Department of Endocrinology, The Second People’s Hospital of Bengbu City, Bengbu, China; 2 Department of Clinical Laboratory, Children’s Hospital, Zhejiang University School of Medicine, National Clinical Research Center for Children and Adolescents’ Health and Diseases, Hangzhou, China; 3 Department of Infectious Diseases, Zhejiang Provincial People’s Hospital, Affiliated People’s Hospital, Hangzhou Medical College, Hangzhou, China

**Keywords:** adrenal venous sampling, cardiovascular risk, primary aldosteronism, risk stratification, steroidomics

## Abstract

Primary aldosteronism (PA) has traditionally been approached as a disorder of anatomical subtype classification, with treatment decisions largely structured around distinction between unilateral and bilateral disease. Although this framework remains essential for therapeutic orientation, it does not fully account for the marked heterogeneity in cardiovascular, renal, and metabolic outcomes observed across patients with PA. Increasing evidence suggests that long-term prognosis in PA reflects not only laterality, but also the cumulative burden of mineralocorticoid receptor activation, current hormonal activity, and interindividual tissue susceptibility. Clinical target-organ damage, persistent renin suppression, magnitude of aldosterone excess, steroidogenic profiles, cortisol co-secretion, and somatic or germline molecular features may each provide complementary information that is not captured by subtype designation alone. In this review, we examine the limitations of a binary anatomical paradigm for risk estimation and summarise emerging evidence supporting a multidimensional framework integrating clinical burden, functional hormonal activity, and molecular context. We further discuss the implications of this framework for treatment adequacy assessment, longitudinal follow-up, and future endpoint-driven research. PA should therefore be considered not only a disorder requiring subtype assignment, but also a stratifiable cardiometabolic condition in which estimation of residual organ risk may be as clinically relevant as determination of anatomical laterality. Prospective validation is still required, but laterality alone appears insufficient for longitudinal risk stratification and treatment adequacy assessment in PA.

## 1 Introduction

Primary aldosteronism (PA) has traditionally been approached within a binary diagnostic and therapeutic framework. In routine practice, screening typically begins with the aldosterone-to-renin ratio (ARR), followed by adrenal computed tomography (CT) and, when appropriate, adrenal venous sampling (AVS) to distinguish unilateral from bilateral aldosterone excess and guide treatment selection. This pathway remains central to contemporary clinical care (1–3). Within this framework, unilateral PA is generally managed with adrenalectomy, whereas bilateral disease is usually treated with mineralocorticoid receptor antagonist (MRA) therapy (2, 3).

However, accumulating evidence suggests that anatomical classification alone does not fully explain interindividual variability in cardiovascular, renal, and metabolic outcomes. Among patients with PA and related forms of renin-independent aldosteronism, organ risk appears heterogeneous and is not consistently determined by laterality alone (4, 5). Population-based physiology studies further suggest that dysregulated aldosterone production may occur across a quantitative spectrum, challenging the binary subtype model as the sole framework for understanding disease burden (6, 7).

These observations do not diminish the importance of conventional subtype evaluation. Rather, they suggest that laterality and prognosis address related but distinct clinical questions in primary aldosteronism (4, 5, 8). Conventional evaluation is primarily intended to establish the diagnosis, determine whether aldosterone excess can be lateralized, and guide the choice between adrenalectomy and medical therapy (1–3). By contrast, long-term management also requires assessment of baseline organ vulnerability, ongoing biological activity, and residual cardiorenal risk after treatment selection (8–10).

**Figure 1:**
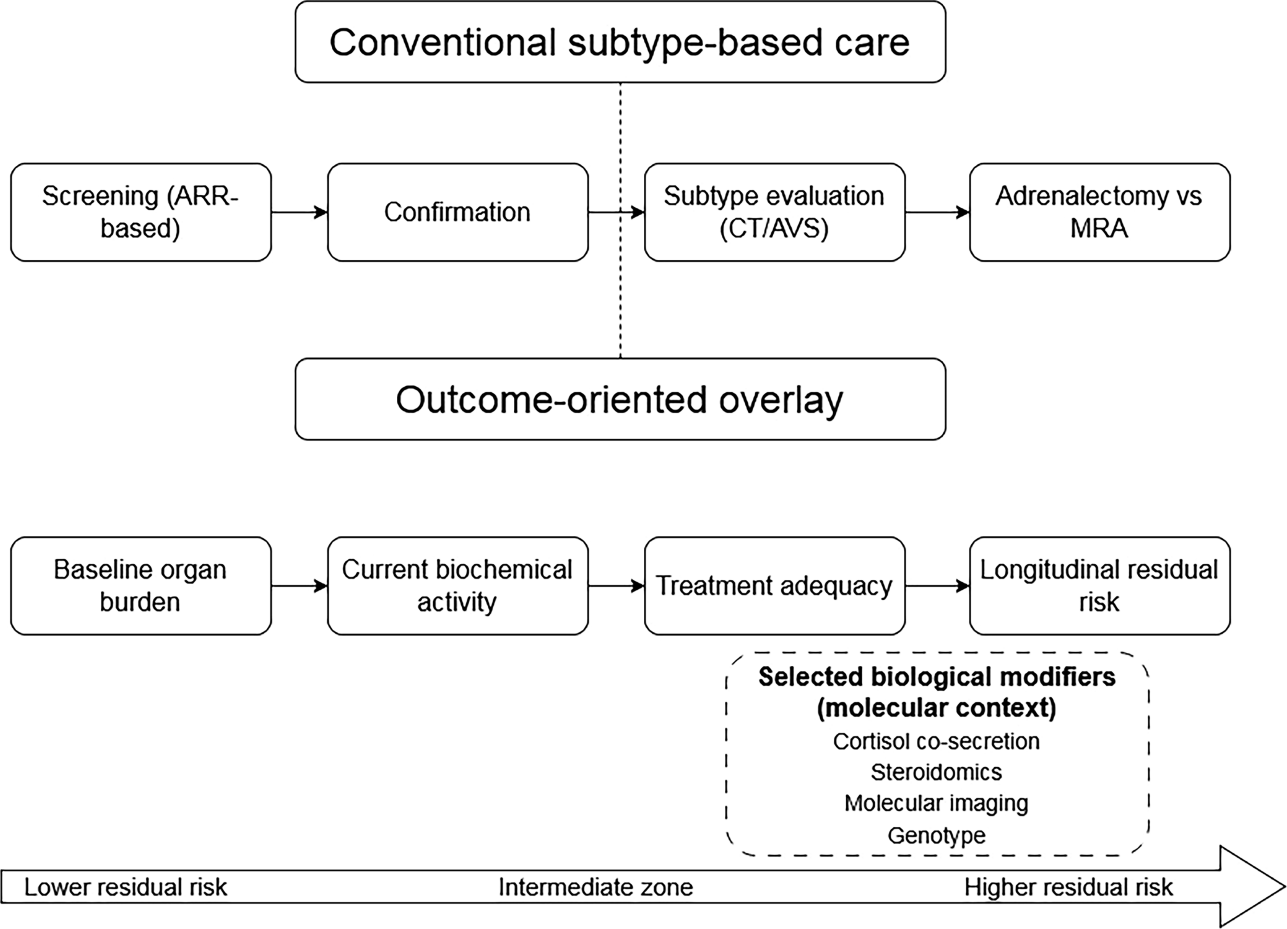
Conventional subtype-based care with an outcome-oriented overlay in primary aldosteronism. The figure illustrates that conventional care in primary aldosteronism remains centred on screening, diagnostic confirmation, subtype evaluation, and treatment selection. The proposed outcome-oriented framework is shown as a complementary overlay, intended not to replace laterality-based care but to refine assessment of baseline organ burden, current biochemical activity, treatment adequacy, residual longitudinal risk, and selected biological modifiers within the broader molecular context. ARR, aldosterone-to-renin ratio; AVS, adrenal venous sampling; CT, computed tomography; MRA, mineralocorticoid receptor antagonist.

Accordingly, this review does not advocate replacement of the conventional pathway. Instead, it examines how an outcome-oriented framework may complement standard screening, diagnostic confirmation, subtype classification, and therapy selection by incorporating three additional dimensions: clinical burden, functional hormonal activity, and molecular context (2, 11, 12). In this formulation, laterality remains central to treatment direction, whereas the broader framework is intended to refine risk estimation and treatment adequacy assessment over time (2, 8, 10). The proposed relationship between these conventional and adjunctive perspectives is summarized in **Figure 1**.

## 2 Methods of evidence identification

This narrative review was informed by a structured literature search of PubMed/MEDLINE, Embase, and Web of Science for English-language articles published up to March 2026. The search focused on primary aldosteronism, laterality, adrenal venous sampling, cardiovascular and renal outcomes, renin dynamics, steroidomics, molecular profiling, and functional imaging. Search terms included both controlled vocabulary and free-text keywords relevant to subtype evaluation, treatment adequacy, and long-term outcome assessment. Priority was given to recent clinical practice guidelines, systematic reviews, meta-analyses, prospective cohort studies, and translational investigations with direct clinical or biological relevance. Landmark earlier studies were also included when necessary to provide conceptual or historical context. As this was a narrative review, the selection and synthesis of evidence were guided by clinical relevance and the contribution of individual studies to the conceptual framework, rather than by a predefined systematic review protocol or formal evidence grading process.

## 3 The conventional subtype-based pathway: strengths, limitations, and unresolved clinical dilemmas

### 3.1 Structural–functional discordance

CT remains the first-line imaging modality for subtype evaluation in PA, but structural findings do not consistently correspond to functional aldosterone secretion. Apparent unilateral adrenal nodules may represent non-functioning incidentalomas, whereas aldosterone excess may arise from a different lesion or from bilateral adrenal tissue (13–15). AVS is therefore commonly used to establish functional lateralisation in patients being considered for adrenalectomy (13, 14). However, AVS is invasive, technically demanding, and not universally available, so reliance on either structural imaging alone or idealised AVS pathways remains problematic in routine clinical practice (13, 14, 16).

### 3.2 Procedural variability and interpretative heterogeneity

AVS remains the reference standard for subtype classification in PA, but its technical success and interpretability vary across centres and depend substantially on operator expertise (13, 16). AVS availability itself remains uneven, with one Australia–New Zealand survey showing provision at only 58% of endocrinology sites, and a recent meta-analysis indicating that intraprocedural cortisol measurement increased bilateral selectivity from 64% to 84% (RR 1.42, 95% CI 1.27–1.59), underscoring that access and technical success remain centre- and protocol-dependent rather than procedure-independent (16–18). Differences in lateralisation criteria and interpretative thresholds can alter subtype assignment and subsequent management (19, 20). Intraprocedural cortisol measurement may improve cannulation success, but it also underscores the procedural dependence of AVS performance (17, 18). Biological factors such as low-grade cortisol co-secretion may modestly affect selected AVS parameters under specific protocols (21). Accordingly, AVS-derived laterality is best interpreted as a high-value functional assessment rather than a completely procedure-independent readout of disease biology (13, 14).

### 3.3 Prognostic limits of the unilateral/bilateral framework

The unilateral-versus-bilateral framework remains clinically pragmatic for treatment selection, but laterality alone does not reliably define long-term cardiovascular or renal risk (8, 9). Postoperative trajectories among patients classified as having unilateral PA remain heterogeneous, long-term recurrence after adrenalectomy has been documented in a subset of patients, and contralateral suppression-based indices have shown inconsistent predictive performance across studies (20, 22–24). Persistent or recurrent biochemical disease has been reported in a minority after adrenalectomy. In one long-term study, recurrence of aldosteronism occurred in 12 of 53 patients (23%) despite initial short-term biochemical remission, while another cohort reported complete biochemical success in 91.1%, partial biochemical success in 7.1%, and absent biochemical success in 1.8% after surgery, reinforcing that postoperative biochemical outcomes are generally favourable but not uniformly durable (22, 23). Anatomical subtype is therefore best interpreted primarily as a treatment-directing framework rather than a comprehensive surrogate for long-term prognosis (8, 9).

## 4 Clinical Evidence for PA as a spectrum of cardiometabolic risk

### 4.1 Cardiovascular risk beyond blood pressure

PA is associated with cardiovascular morbidity that exceeds that expected from blood pressure elevation alone and is often greater than that observed in comparable essential hypertension. Meta-analyses have consistently linked PA to more pronounced left ventricular remodelling, a higher prevalence of atrial fibrillation, and an increased risk of stroke (5, 25, 26). Earlier meta-analytic evidence has further suggested that, compared with essential hypertension, PA is associated with materially higher risk of major cardiovascular outcomes, including stroke (OR 2.50, 95% CI 2.08–3.02) and atrial fibrillation (OR 3.17, 95% CI 2.09–4.80), underscoring that the excess burden is clinically substantial rather than marginal (25, 26). Correction of aldosterone excess attenuates, but does not uniformly eliminate, this excess risk. Both adrenalectomy and MRA therapy can improve left ventricular remodelling and reduce subsequent cardiovascular risk, although residual vulnerability may persist in patients with prolonged exposure or established structural injury (5, 26, 27). These observations suggest that cardiovascular risk in PA reflects cumulative hormonal burden and target-organ susceptibility rather than blood pressure alone.

### 4.2 Renal trajectory

PA is commonly characterised by relative glomerular hyperfiltration driven by sodium retention and intraglomerular hypertension. This may mask underlying structural renal injury and lead to overestimation of baseline renal function. After correction of aldosterone excess, an early decline in estimated glomerular filtration rate (eGFR) is frequently observed and is generally interpreted as reversal of hyperfiltration rather than treatment-related nephrotoxicity (28, 29). The magnitude of this initial eGFR dip has been associated with subsequent renal trajectory in some studies, suggesting that it may partly reflect antecedent hormonal burden and previously unmasked renal vulnerability (29). Albuminuria is also more common in PA than in essential hypertension and often decreases after targeted treatment (5, 30). Overall, the renal course in PA is dynamic, and post-treatment renal recovery varies across individuals.

### 4.3 Metabolic and systemic effects

PA has also been associated with greater insulin resistance and disordered glucose metabolism than essential hypertension (5, 31, 32). Although effect sizes vary across studies, metabolic dysfunction appears to be a reproducible component of the PA phenotype. Experimental and translational evidence further suggests that mineralocorticoid receptor (MR) signalling in metabolically active tissues may contribute to impaired insulin sensitivity and inflammatory activation (33). Some glucose-related parameters improve after correction of aldosterone excess, although the available evidence remains largely observational and heterogeneous (11, 32).

### 4.4 Continuum of renin-independent aldosterone excess

Population-based physiological studies suggest that suppressed renin with relatively increased aldosterone can be detected across a broad spectrum of blood pressure phenotypes beyond overt PA (6, 7). Histopathological and immunohistochemical studies have also identified aldosterone-producing micronodules in adrenal glands without overt tumoural lesions, some of which harbour somatic mutations similar to those found in aldosterone-producing adenomas (34, 35). These observations support the view that autonomous aldosterone production may exist along a biological continuum rather than within a strictly binary anatomical model.

### 4.5 Genetic heterogeneity and phenotypic diversity

Somatic mutations involving ion channels and ATPase-related pathways are common in aldosterone-producing adenomas and are associated with distinct steroidogenic signatures (35–37). Mutation-specific effects on membrane depolarisation, calcium signalling, and CYP11B2 expression help to explain variation in aldosterone secretion and hybrid steroid profiles (35, 36). Genotype–phenotype correlations further suggest that molecular substrate contributes to differences in hormonal burden and clinical presentation, although associations with long-term cardiovascular or renal outcomes remain incompletely defined (37, 38).

## 5 Biological basis of heterogeneity: tissue-specific MR signalling and modifiers of risk

### 5.1 Organ-specific MR activation and shared remodelling pathways

Aldosterone excess is the defining hormonal abnormality in PA, but its downstream effects depend not only on circulating concentration but also on tissue-specific MR activation, sodium milieu, redox state, and intrinsic organ susceptibility (12, 39). In the distal nephron, MR signalling promotes sodium reabsorption, potassium excretion, and volume expansion, thereby contributing centrally to hypertension. These classical renal actions do not fully explain the broader cardiovascular and metabolic phenotype (12, 40). In non-epithelial tissues, including the heart, vasculature, kidney, adipose tissue, and immune compartments, MR activation contributes to oxidative stress, inflammatory signalling, fibrosis, endothelial dysfunction, arterial stiffening, myocardial hypertrophy, and renal injury (39, 41–46). Organ injury in PA is therefore better understood as the integrated result of hormonal burden acting on tissues with variable susceptibility rather than as a simple linear consequence of circulating aldosterone alone.

### 5.2 Modifiers of the risk gradient

Patients with similar aldosterone concentrations may nonetheless follow markedly different clinical trajectories because tissue response is shaped by contextual factors that amplify or attenuate MR-dependent injury.

Dietary sodium is a major amplifier of aldosterone-mediated damage. Experimental, physiological, and clinical evidence supports a high-salt environment as a potentiator of MR-dependent inflammation, oxidative stress, and fibrotic remodelling, particularly in cardiovascular and renal tissues (40, 47, 48). This interaction is clinically relevant in PA, in which excess sodium exposure may intensify target-organ injury even when aldosterone concentrations are not extreme (10, 49).

Cortisol co-secretion is another potential modifier. Combined glucocorticoid and mineralocorticoid excess may aggravate metabolic dysfunction and cardiovascular remodelling beyond that seen with isolated aldosterone excess, although the available clinical evidence remains limited and heterogeneous (50, 51).

Genetic and molecular substrate may further influence hormonal output and downstream signalling. Somatic driver mutations alter ion-channel behaviour, calcium signalling, steroidogenic enzyme expression, and aldosterone biosynthesis, thereby contributing to heterogeneity in biological activity (34, 52, 53). Genotype-associated differences have been reported, particularly in KCNJ5-associated disease, but they are not sufficiently consistent to function as standalone prognostic classifiers (36–38). Emerging spatial and molecular studies further suggest that intratumoral and inter-nodular heterogeneity may shape steroidogenic behaviour beyond conventional subtype labels (53–55).

Systemic hormonal context also modifies risk. Renin-independent aldosteronism has been associated with greater cardiovascular burden than renin-dependent states, suggesting that autonomous MR pathway activation has implications beyond blood pressure elevation alone (4, 5).

## 6 Functional phenotyping beyond ARR: steroidomics and molecular imaging

### 6.1 From ARR to multidimensional steroid profiling

ARR remains the standard screening tool for PA, but it provides limited insight into the broader architecture of adrenal steroidogenesis. Its interpretation is influenced by posture, medication exposure, sodium balance, and assay characteristics (1, 3). Multidimensional steroid profiling using liquid chromatography–tandem mass spectrometry (LC–MS/MS) enables simultaneous quantification of mineralocorticoids, glucocorticoids, and precursor steroids, thereby extending functional resolution beyond single-hormone measurement (56, 57). Hybrid steroids such as 18-oxocortisol and 18-hydroxycortisol are often enriched in aldosterone-producing adenomas and may reflect altered steroidogenic flux, particularly in settings of dysregulated CYP11B2 activity and altered pathway coupling (58, 59). By capturing coordinated steroidogenic patterns rather than isolated aldosterone concentrations, steroidomics may provide complementary information on the intensity and pattern of hormone production.

### 6.2 Steroid signatures and clinical relevance

Beyond subtype characterisation, steroidomic patterns may also have potential clinical relevance. Elevated hybrid steroids and selected precursor patterns have been associated with greater aldosterone biosynthetic activity and, in exploratory studies, with biologically more active disease phenotypes (58–60). Preoperative steroid profiles have also been linked to biochemical outcomes after adrenalectomy in selected cohorts, raising the possibility that steroidomics may contribute to preoperative estimation of treatment response (61). However, these findings remain heterogeneous across cohorts and analytical platforms, and the role of steroidomics in routine clinical risk stratification remains provisional.

### 6.3 Translational constraints and standardization requirements

Clinical integration of steroidomic profiling remains constrained by methodological variability and limited accessibility. Differences in analytical platforms, calibration procedures, sample preparation, and pre-analytical handling may contribute to inter-laboratory heterogeneity (56, 57). Standardised reference intervals, quality-control procedures, and reporting frameworks will be essential for broader adoption (56, 57). Prospective multicentre validation is needed to determine whether steroidomic data provide clinically useful information beyond established diagnostic pathways (57, 62).

### 6.4 Molecular imaging as an emerging adjunct to functional subtyping

Recent advances in molecular imaging are also relevant to efforts to complement conventional cross-sectional imaging and adrenal venous sampling (AVS)-based subtyping in primary aldosteronism (13, 62, 63). Functional imaging approaches, particularly ^11C-metomidate positron emission tomography-computed tomography (PET-CT), and, more recently, emerging CYP11B2-directed tracers, aim to identify steroidogenically active adrenal tissue rather than structural abnormality alone (62, 63). In selected settings, these methods may provide complementary information on functional lateralization, help resolve discordance between imaging and biochemistry, and offer additional diagnostic support when AVS is unavailable, unsuccessful, or difficult to interpret (13, 62, 63). Notably, the prospective within-patient MATCH trial enrolled 143 patients with PA, of whom 128 reached 6- to 9-month follow-up, providing some of the strongest direct comparative data for ^11C-metomidate PET-CT against AVS in this setting (64).

However, their current clinical role remains complementary rather than definitive. Availability is limited, protocols are not yet standardized across centres, and outcome-based validation remains less mature than for established subtype pathways, particularly for newer CYP11B2-directed tracers (62, 63). Accordingly, molecular imaging should currently be viewed as a promising adjunct for selected patients and specialized centres, rather than as a routine replacement for AVS (13, 63). Its main conceptual relevance to the present framework is that it links anatomical localization with biological activity and may therefore refine, rather than replace, conventional subtype-based decision-making (62, 63).

## 7 Genotype and molecular subtyping: biological insight and predictive boundaries

### 7.1 Somatic mutations and surgical outcomes

Aldosterone-producing adenomas frequently harbour recurrent somatic mutations affecting calcium signalling and steroidogenic output (36, 37, 53). Certain mutation classes, particularly KCNJ5, have been associated with higher aldosterone production and distinct biochemical profiles (36, 38). Observational studies further suggest that KCNJ5-mutated disease may be associated with a higher likelihood of biochemical remission and, in some cohorts, better hypertension improvement after adrenalectomy (36, 38, 65), whereas for other mutation subtypes the associations with surgical response remain less consistent (53). However, postoperative blood pressure outcomes remain strongly influenced by non-genetic factors, including hypertension duration, age, baseline vascular remodelling, and antihypertensive treatment burden (2, 37). Prospective data linking genotype to long-term cardiovascular or renal outcomes remain limited, although recent systematic review evidence suggests potential differences in cardiovascular recovery in KCNJ5-mutated aldosterone-producing adenomas (66). Because genotyping usually relies on postoperative tumour tissue, its preoperative utility remains restricted. Somatic mutation status therefore provides important biological context but does not independently determine treatment selection in current practice.

### 7.2 Germline variants and familial disease trajectory

Familial forms of PA arise from germline variants affecting ion-channel function, calcium signalling, or steroidogenic regulation (67, 68). These disorders often present at a younger age, potentially prolonging cumulative hormonal exposure. Phenotypic expression varies across genotypes, and genotype–phenotype correlations remain incompletely defined (67, 68). Identification of pathogenic variants informs family screening and earlier biochemical evaluation in at-risk relatives, but treatment selection still depends primarily on functional subtype and clinical context (67, 68). Specific defects involving calcium-channel genes such as CACNA1H further illustrate the molecular heterogeneity of familial PA (69).

### 7.3 Integrating genotype and steroidomics in subtype prediction

Integration of molecular and biochemical data has been proposed as a probabilistic approach to subtype estimation in PA. Mutation-associated biochemical signatures captured by multidimensional steroid profiling may improve discrimination between unilateral and bilateral disease in exploratory models (70). However, most currently available algorithms have been derived from retrospective cohorts and remain insufficiently validated across external populations. Although combined clinical–biochemical–molecular models may support individualised risk discussion in selected settings, they do not replace established lateralisation procedures when definitive surgical decisions are required (2). At present, molecular and steroidomic data are best regarded as adjunctive tools for biological characterisation rather than standalone determinants of clinical management.

## 8 Assessing treatment adequacy: beyond blood pressure control

### 8.1 Renin as a functional marker

In PA, treatment response has traditionally been assessed by normalisation of blood pressure and correction of hypokalaemia. Although clinically important, these endpoints do not necessarily capture residual MR pathway activity. Renin suppression is a central feature of autonomous aldosterone excess, and restoration of renin from suppressed to detectable or unsuppressed levels during MRA therapy has been associated with more favourable cardiorenal profiles in observational studies and recent meta-analytic evidence (10, 71). This suggests that renin recovery may reflect more effective suppression of persistent sodium-retentive physiology and MR pathway activity.

However, routine renin-guided titration remains limited by methodological and clinical uncertainty. No universally accepted renin threshold has been established to define adequate MR blockade, and renin measurements are influenced by assay variability, sodium intake, posture, and concomitant medication (3, 72). A recent systematic review and meta-analysis including 24 studies and 6621 medically treated patients found that, in the primary meta-analysis, unsuppressed post-treatment renin was associated with a lower risk of cardiovascular events than suppressed renin (pooled HR 0.43, 95% CI 0.23–0.80), supporting its use as a pragmatic marker of residual biological activity and treatment adequacy rather than a rigid universal threshold (71). Available outcome data also derive largely from retrospective analyses, with limited prospective validation. Renin is therefore best interpreted as a dynamic functional marker that complements, rather than replaces, clinical assessment.

### 8.2 Biomarkers of residual MR activity

Interest has increasingly shifted towards circulating and urinary biomarkers that may reflect persistent MR pathway activation beyond conventional haemodynamic measures. Candidate indicators explored to date include circulating biomarkers related to extracellular matrix turnover, Galectin-3, selected microRNA profiles, and urinary extracellular vesicle-derived proteins related to distal tubular sodium handling, including γENaC (73–76). However, most available evidence remains associative, derives from small or single-centre cohorts, and lacks validated clinical thresholds. These biomarkers should therefore still be regarded as investigational.

### 8.3 Future directions

Direct quantification of tissue-level MR activity remains difficult in clinical practice. Emerging imaging, transcriptomic, proteomic, and metabolomic approaches may improve assessment of tissue remodelling and organ-level response to therapy (12, 77). At present, however, these methods remain exploratory adjuncts rather than established components of routine clinical assessment.

## 9 Outcome-oriented risk stratification framework

### 9.1 A complementary three-domain framework across stages of care

The proposed framework is not intended to displace the conventional diagnostic pathway. Rather, it adds a complementary clinical perspective to established care. Conventional evaluation addresses whether primary aldosteronism is present, whether aldosterone excess can be lateralized, and whether adrenalectomy or medical therapy is the appropriate treatment (2, 3). Outcome-oriented assessment addresses a different but related set of questions: how much target-organ injury is already present, how much autonomous biological activity persists, and whether clinically relevant residual risk remains despite apparently adequate treatment (4, 5, 10). The three domains, their representative variables, and their current degree of clinical maturity are summarized in **Table 1**.

**Table 1:** Proposed multidimensional domains for outcome-oriented risk stratification in primary aldosteronism.

Domain	Representative variables	What the domain reflects	Clinical maturity
Clinical burden	LV remodelling; albuminuria/renal dysfunction; hypertension duration; cardiometabolic comorbidity	Accumulated target-organ consequences of chronic aldosterone excess and baseline vulnerability	Clinically accessible; relevant to routine risk assessment
Functional hormonal activity	Persistent renin suppression; renin recovery during therapy; aldosterone excess; biochemical treatment response; steroidomic profile	Current biological activity and adequacy of hormonal control beyond blood pressure alone	Partly translatable to practice; interpretation may vary by treatment status and assay context
Molecular context	Somatic driver mutations; genotype-dependent morphology; candidate molecular markers	Mechanistic heterogeneity influencing aldosterone production pattern and possibly long-term risk	Mainly adjunctive or investigational at present

This table summarises three complementary domains—clinical burden, functional hormonal activity, and molecular context—that may refine long-term risk stratification in primary aldosteronism beyond anatomical laterality alone. Representative variables, their conceptual implications, and their current degree of clinical maturity are shown. LV, left ventricular.

In practical terms, the three domains can be operationalized with different levels of clinical maturity. The clinical domain is the most immediately translatable and includes variables already available in routine care, such as hypertension duration, antihypertensive treatment burden, hypokalaemia, left ventricular remodelling when assessed, atrial fibrillation, albuminuria, and renal function (11, 25, 27). The functional domain includes the degree of renin suppression, the magnitude of aldosterone excess, biochemical response after adrenalectomy, and renin recovery during mineralocorticoid receptor antagonist therapy (10, 71, 78). The molecular domain currently remains more limited in routine practice and includes cortisol co-secretion, mutation-associated biology, steroidomic patterns, and selected functional imaging findings where available (50, 56, 63). In this formulation, the first two domains are relevant to most centres, whereas the third is primarily adjunctive (62, 63).

### 9.2 A risk continuum across biological burden and organ vulnerability

Patients with PA may be distributed along a graded continuum of biological activity and organ vulnerability (6, 7). At one end are patients without clear structural organ involvement and without evidence of sustained hormonal activity during treatment. At the other are patients with persistent biochemical activity and established cardiac or renal remodelling, in whom cumulative biological burden may be greater irrespective of anatomical subtype (10, 71). Between these extremes lies a broad intermediate zone that probably includes many patients encountered in routine practice. In such cases, longitudinal reassessment of biochemical markers, organ involvement, and clinical trajectory may be more informative than reliance on cross-sectional categorisation alone, particularly when biochemical indices are influenced by medication exposure or measurement variability (78, 79).

### 9.3 Potential therapeutic implications of risk-oriented assessment

Within this framework, treatment selection still begins with standard subtype-based care. Laterality remains central to the choice between adrenalectomy and medical therapy (2, 3). What changes is not the basic treatment algorithm, but the depth of baseline phenotyping and the structure of follow-up after treatment has been initiated (2, 3).

For example, patients managed medically may benefit from reassessment that considers persistent renin suppression, renal trajectory, albuminuria, and evidence of target-organ involvement in addition to blood pressure and potassium (51, 71, 78). Likewise, patients after adrenalectomy may still warrant longitudinal surveillance when pre-existing organ damage is substantial, biochemical recovery is incomplete, or uncertainty regarding long-term remission persists (23, 25, 30).

Thus, outcome-oriented assessment is best understood as a refinement of treatment adequacy assessment and longitudinal follow-up, rather than as a replacement for conventional decision-making (2, 3). In practical terms, escalation beyond conventional follow-up may be most relevant in patients with substantial baseline target-organ damage, persistent renin suppression during medical therapy, incomplete biochemical recovery after adrenalectomy, or discordance between apparent subtype control and subsequent clinical trajectory (23, 71, 78). Its value lies in identifying patients whose residual cardiorenal risk may remain clinically relevant even after subtype-appropriate therapy has been selected (23, 51, 71).

### 9.4 A longitudinal and adaptive perspective

Risk in PA is unlikely to be static. Baseline assessment may be informed by integrated evaluation of organ status and markers of pathway activity, followed by periodic reassessment of renal function, cardiac structure, and biochemical response over time (10). Emerging diagnostic approaches, including optimised AVS practice and molecular imaging, may further support individualised evaluation in selected settings, although these strategies remain variably validated (62, 63).

## 10 Clinical implementation and research priorities

### 10.1 Interpretive considerations across resource settings

In primary care or resource-limited settings, structured assessment using ARR, potassium, clinical phenotype, and adrenal imaging when available may help identify patients in whom PA is sufficiently likely to warrant further evaluation or targeted treatment (1, 80). Secondary-level centres may integrate clinical features, biochemical findings, evidence of target-organ involvement, and imaging concordance to refine probabilistic interpretation. Tertiary centres are best positioned to undertake more comprehensive evaluation, including AVS and selected advanced phenotyping, particularly when clarification of laterality is expected to alter management (2, 3). A tiered perspective may therefore help align diagnostic complexity with anticipated clinical relevance. In addition to centre-level resource constraints, patient-level factors such as affordability, access to specialist care, health literacy, and treatment preferences may also influence the feasibility of extended phenotyping and longitudinal monitoring (80–82). In practical terms, implementation may proceed stepwise. First, clinicians should prioritise scalable markers already obtainable in most settings, including blood pressure phenotype, potassium, renal function, albuminuria when available, and renin status where interpretable (3, 79). Second, patients with substantial target-organ burden, persistent renin suppression during medical therapy, discordance between imaging and biochemistry, or incomplete biochemical recovery after adrenalectomy may be prioritised for intensified follow-up or referral (22, 70). Third, advanced adjuncts such as steroidomics, molecular imaging, and broader molecular profiling are best reserved for selected tertiary centres or prospective research, particularly when they are likely to clarify unresolved biological heterogeneity or materially alter management (61, 62). A pragmatic tiered implementation of this framework across different healthcare settings is outlined in **Table 2**. The distinct but complementary roles of conventional subtype-based care and outcome-oriented assessment across different stages of management are summarized in **Table 3**.

**Table 2:** Pragmatic implementation of an outcome-oriented framework across healthcare settings.

Setting/purpose	Core assessment components	Potential added value beyond laterality	Main limitations
Primary care/initial evaluation	ARR; potassium; clinical phenotype; blood pressure profile; adrenal imaging when available	Identification of patients with overt organ burden or high likelihood of renin-independent aldosterone excess	Limited access to AVS, steroidomics, and advanced phenotyping
Secondary-level care	ARR; imaging review; renal function; markers of target-organ damage; treatment response	Risk enrichment using clinical burden and biochemical persistence to guide referral and intensification	Subtype uncertainty often remains without AVS
Tertiary referral care	AVS; structured cardiac and renal assessment; optimized MRA titration; selected steroidomic or molecular adjuncts	Multidimensional phenotyping for subtype clarification, treatment adequacy assessment, and longitudinal follow-up	Greater complexity, cost, and limited standardization of adjunctive markers
Research setting	Prospective cardiovascular and renal endpoints; standardized biochemical response metrics; scalable phenotyping strategies	Validation of integrated models beyond anatomical classification alone	Requires multicentre validation and demonstration of clinical utility

The table summarises how an outcome-oriented framework in primary aldosteronism may be applied across different healthcare settings, from initial evaluation to tertiary referral and research contexts. It emphasizes the potential role of staged assessment beyond anatomical laterality, while acknowledging practical limitations related to access, complexity, standardization, and validation. ARR, aldosterone-to-renin ratio; AVS, adrenal venous sampling; CT, computed tomography; MRA, mineralocorticoid receptor antagonist.

**Table 3:** Conventional subtype-based care and outcome-oriented assessment address distinct but complementary clinical questions across the primary aldosteronism care pathway.

Stage of care	Main question in conventional subtype-based care	Core conventional tools	Additional question in an outcome-oriented approach	Most feasible adjuncts in current practice	Advanced or selected adjuncts
Screening/initial suspicion	Could this patient have primary aldosteronism?	Aldosterone-to-renin ratio; aldosterone and renin measurements; potassium; clinical and blood pressure phenotype	Is there already evidence of clinically relevant organ vulnerability or a high-risk phenotype at presentation?	Hypertension duration; antihypertensive treatment burden; hypokalaemia; basic renal function; albuminuria when available	Structured cardiovascular phenotyping in selected patients
Diagnostic confirmation/initial phenotyping	Is autonomous aldosterone excess likely present?	Repeat biochemical testing; confirmatory testing where appropriate; adrenal CT as part of initial evaluation	How biologically active and clinically mature is the disease beyond biochemical confirmation alone?	Degree of renin suppression; severity of hypertension; baseline renal function; albuminuria; evidence of target-organ involvement	Assessment of cortisol co-secretion in selected cases
Subtype evaluation/treatment selection	Is disease lateralized, and should treatment be adrenalectomy or mineralocorticoid receptor antagonist therapy?	Adrenal CT; adrenal venous sampling in appropriate surgical candidates	Which patients may remain at higher residual risk regardless of treatment pathway?	Baseline cardiac and renal injury; comorbidity burden; renal reserve; biochemical severity	Steroidomics; molecular imaging; selected molecular profiling in specialized centres
Early post-treatment assessment	Has treatment corrected the immediate biochemical and clinical manifestations?	Blood pressure; potassium; postoperative biochemical outcome or response to mineralocorticoid receptor antagonist therapy	Is residual mineralocorticoid receptor pathway activity still likely despite apparent initial control?	Renin response during medical therapy; early eGFR trajectory; albuminuria trend; persistence of antihypertensive requirement	Investigational biomarkers of residual mineralocorticoid receptor activity
Long-term follow-up	Is there persistent hypertension, recurrence, treatment intolerance, or need for regimen adjustment?	Clinical review; blood pressure monitoring; potassium; renal function; repeat biochemical assessment as indicated	Does clinically meaningful cardiovascular, renal, or metabolic risk remain despite subtype-appropriate treatment?	Longitudinal renal trajectory; atrial fibrillation surveillance when clinically indicated; assessment of target-organ damage over time	Advanced imaging, omics, or biomarker-guided monitoring in selected centres or research settings

This table illustrates that conventional subtype-based care and outcome-oriented assessment are complementary rather than competing approaches in primary aldosteronism. Conventional care remains essential for diagnosis, subtype assignment, and treatment selection, whereas outcome-oriented assessment may refine baseline phenotyping, treatment adequacy assessment, and estimation of residual long-term cardiorenal risk. CT, computed tomography; eGFR, estimated glomerular filtration rate.

### 10.2 Trial design: from surrogate control to hard endpoints

Interventional evidence in PA has largely focused on blood pressure improvement, correction of hypokalaemia, and biochemical normalisation. Future prospective and, where feasible, randomised studies should increasingly evaluate clinically meaningful cardiovascular and renal outcomes, including atrial fibrillation, heart failure hospitalisation, stroke, and chronic kidney disease progression, to determine whether targeted interventions confer durable risk reduction (8, 71). Intermediate markers such as left ventricular mass, albuminuria, and renal function trajectory may serve as supportive endpoints, but should be interpreted as surrogates rather than substitutes for event-based outcomes (9, 27). Risk-enriched enrolment strategies may improve feasibility by preferentially including patients with established target-organ involvement or persistent biological activity (3, 71).

### 10.3 Economic and health system considerations

More stratified assessment may increase initial diagnostic complexity, resource use, and cost, particularly when advanced biochemical, imaging, or procedural tools are incorporated (62, 80). Whether such strategies improve longer-term efficiency will depend on their ability to identify patients in whom changes in management translate into meaningful reductions in cardiovascular or renal events. Formal economic evaluation will therefore be important to determine the incremental value of tiered assessment across different healthcare settings (3, 80).

### 10.4 Current evidence gaps and methodological constraints

Despite increasing interest in outcome-oriented risk stratification in primary aldosteronism, the current evidence base remains methodologically uneven. Much of the available literature is retrospective, single-centre, or based on surrogate endpoints rather than hard cardiovascular or renal outcomes, and there is substantial heterogeneity in screening strategies, confirmatory testing, adrenal venous sampling protocols, assay platforms, and treatment targets (62, 72). These differences complicate cross-study comparison and limit the immediate generalizability of proposed risk markers.

In addition, many emerging tools, including steroidomic signatures, molecular predictors, and biomarkers of residual mineralocorticoid receptor activity, remain variably standardized and are not yet routinely accessible across healthcare settings. Even when biologically informative, their incremental value beyond conventional clinical assessment has not been consistently validated in prospective endpoint-driven studies.

Accordingly, current multidimensional models should be interpreted as a conceptual and hypothesis-generating framework rather than a validated clinical algorithm. Future studies should prioritize prospective validation, harmonized definitions of treatment adequacy, and assessment of whether integrated models improve prediction of cardiovascular, renal, and metabolic outcomes beyond laterality-based classification alone.

## 11 Discussion

This review argues that primary aldosteronism should not be interpreted solely through anatomical laterality when the clinical objective extends beyond treatment allocation to longitudinal risk estimation and treatment adequacy assessment (8, 10, 71). At the same time, the conventional pathway remains indispensable. Screening with aldosterone-renin measurements, confirmatory evaluation where appropriate, and subtype-oriented treatment selection continue to form the foundation of clinical care (2, 3). In this sense, laterality is not displaced by an outcome-oriented approach; rather, it is complemented by additional assessment of target-organ burden, biochemical activity, and selected biological modifiers (4, 5, 63).

A central implication is that conventional and outcome-oriented approaches answer different but interrelated questions. Conventional care primarily addresses diagnosis, subtype assignment, and the choice between adrenalectomy and medical therapy (2, 3). Outcome-oriented phenotyping may help clarify how much residual cardiovascular, renal, or metabolic risk remains before and after treatment selection (10, 25, 27). At present, the most clinically translatable elements of such an approach are not molecular tests, but rather structured assessment of target-organ damage, renal trajectory, aldosterone-renin dynamics, and treatment response over time, particularly in routine clinical settings (10, 11, 71). Accordingly, current implementation should prioritize scalable markers that are already obtainable in most practices, whereas steroidomics, molecular imaging, and broader molecular profiling should currently be reserved for selected tertiary settings or prospective research (56, 62, 63). The most pragmatic priority is therefore not universal deployment of advanced molecular tools, but more consistent incorporation of scalable clinical and biochemical markers—particularly target-organ assessment, renal trajectory, and renin response—into baseline phenotyping and follow-up.

The evidence base nevertheless remains incomplete. Much of the current literature is observational, definitions and treatment targets vary across studies, and several promising tools—including steroidomics, molecular imaging, and biomarkers of residual mineralocorticoid receptor activity—are not yet standardized for routine use (56, 62, 63). For that reason, the proposed framework should be interpreted as a pragmatic and hypothesis-generating extension of current care rather than as a validated replacement for guideline-directed management (3, 62).

Taken together, laterality should remain central to therapeutic orientation, but it is unlikely to be sufficient for comprehensive longitudinal risk assessment in all patients with primary aldosteronism (8, 23, 71). Future work should therefore focus on validating scalable models that integrate clinical burden, functional hormonal activity, and selected biological modifiers, with particular attention to whether such models improve prediction of cardiovascular and renal outcomes, treatment monitoring, and clinical decision-making beyond subtype classification alone (62, 63, 71).
